# Amide proton transfer-weighted CEST MRI for radiotherapy target delineation of glioblastoma: a prospective pilot study

**DOI:** 10.1186/s41747-024-00523-4

**Published:** 2024-10-30

**Authors:** Patrick L. Y. Tang, Alejandra Méndez Romero, Remi A. Nout, Caroline van Rij, Cleo Slagter, Annemarie T. Swaak-Kragten, Marion Smits, Esther A. H. Warnert

**Affiliations:** 1 Brain Tumor Center, Erasmus MC Cancer Institute, University Medical Center Rotterdam, Rotterdam, The Netherlands; 2 Department of Radiotherapy, Erasmus MC Cancer Institute, University Medical Center Rotterdam, Rotterdam, The Netherlands; 3 Department of Radiology & Nuclear Medicine, Erasmus MC, University Medical Center Rotterdam, Rotterdam, The Netherlands; 4 Medical Delta, Delft, The Netherlands

**Keywords:** Glioblastoma, Magnetic resonance imaging, Neuroimaging, Radiotherapy, Radiotherapy (image-guided)

## Abstract

**Background:**

Extensive glioblastoma infiltration justifies a 15-mm margin around the gross tumor volume (GTV) to define the radiotherapy clinical target volume (CTV). Amide proton transfer (APT)-weighted imaging could enable visualization of tumor infiltration, allowing more accurate GTV delineation. We quantified the impact of integrating APT-weighted imaging into GTV delineation of glioblastoma and compared two APT-weighted quantification methods—magnetization transfer ratio asymmetry (MTR_asym_) and Lorentzian difference (LD) analysis—for target delineation.

**Methods:**

Nine glioblastoma patients underwent an extended imaging protocol prior to radiotherapy, yielding APT-weighted MTR_asym_ and LD maps. From both maps, biological tumor volumes were generated (BTV_MTRasym_ and BTV_LD_) and added to the conventional GTV to generate biological GTVs (GTV_bio,MTRasym_ and GTV_bio,LD_). Wilcoxon signed-rank tests were performed for comparisons.

**Results:**

The GTV_bio,MTRasym_ and GTV_bio,LD_ were significantly larger than the conventional GTV (*p* ≤ 0.022), with a median volume increase of 9.3% and 2.1%, respectively. The GTV_bio,MTRasym_ and GTV_bio,LD_ were significantly smaller than the CTV (*p* = 0.004), with a median volume reduction of 72.1% and 70.9%, respectively. There was no significant volume difference between the BTV_MTRasym_ and BTV_LD_ (*p* = 0.074). In three patients, BTV_MTRasym_ delineation was affected by elevated signals at the brain periphery due to residual motion artifacts; this elevation was absent on the APT-weighted LD maps.

**Conclusion:**

Larger biological GTVs compared to the conventional GTV highlight the potential of APT-weighted imaging for radiotherapy target delineation of glioblastoma. APT-weighted LD mapping may be advantageous for target delineation as it may be more robust against motion artifacts.

**Relevance statement:**

The introduction of APT-weighted imaging may, ultimately, enhance visualization of tumor infiltration and eliminate the need for the substantial 15-mm safety margin for target delineation of glioblastoma. This could reduce the risk of radiation toxicity while still effectively irradiating the tumor.

**Trial registration:**

NCT05970757 (ClinicalTrials.gov).

**Key Points:**

- Integration of APT-weighted imaging into target delineation for radiotherapy is feasible.
- The integration of APT-weighted imaging yields larger GTVs in glioblastoma.
- APT-weighted LD mapping may be more robust against motion artifacts than APT-weighted MTR_asym_.

**Graphical Abstract:**

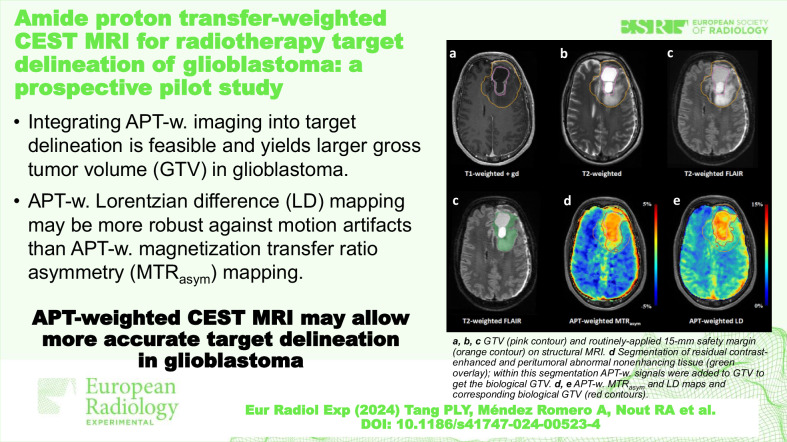

## Background

Glioblastoma is the most common type of primary brain cancer and is generally associated with a poor prognosis [1, 2]. One of the pillars of glioblastoma treatment is radiotherapy, where ionizing radiation is aimed toward a specific target area within the brain to kill tumor cells and slow further tumor growth. To define this target area, a gross tumor volume (GTV) is first delineated on a combination of structural magnetic resonance imaging (MRI) scans, comprising contrast-enhanced T1-weighted, T2-weighted, and T2-weighted fluid-attenuated inversion recovery (FLAIR) sequences [3]. In glioblastoma, the GTV is routinely defined by the resection cavity plus residual enhancing tumor on contrast-enhanced T1-weighted MRI, without the inclusion of peritumoral nonenhancing tissue abnormalities [3]. The exclusion of this area, typically hyperintense on T2-weighted and T2-weighted FLAIR MRI, remains a subject of controversy, given the understanding that these regions may partially reflect tumor infiltration together with edema [4]. After delineation of GTV, the clinical target volume (CTV) is generated by adding a safety margin to the GTV, adjusted for anatomical barriers. This safety margin serves the purpose of accounting for tumor infiltration which is not fully visible on structural MRI. As glioblastomas are notorious for extensive tumor infiltration, the CTV margin is typically 15 mm in every direction [3]. This 15-mm margin effectively covers tumor infiltration in the vast majority of cases, but can also result in large target areas that include a considerable amount of healthy tissue [5–8]. This can, in turn, lead to (severe) radiation-induced side effects, like cognitive impairment, headache, nausea, and fatigue, and substantially decrease the quality of life of a patient [9].

An opportunity to indirectly visualize tumor infiltration emerges with amide proton transfer (APT) weighted imaging, a recently introduced MRI technique that probes local levels of endogenous mobile proteins and peptides through chemical exchange saturation transfer (CEST) [10]. Elevated APT-weighted signal is correlated with increased Ki-67 expression and cell density in human gliomas, highlighting the potential to identify regions with active tumor proliferation [11–13]. Hence, the inclusion of biological information from APT-weighted imaging could enhance the accuracy of GTV delineation of glioblastoma and may obviate the need for the substantial 15-mm CTV margin [14]. This proposition could reduce the risk of radiation-induced side effects while still adequately targeting viable tumor infiltration. Quantification of the APT-weighted CEST effects is commonly determined through the APT-weighted magnetization transfer ratio asymmetry (MTR_asym_) [15]. This approach is valued for its relative simplicity, and only necessitates the acquisition of the CEST effect at two off-resonance frequency shifts (3.5 ppm and -3.5 ppm), and few offsets for B_0_ correction. In contrast, APT-weighted Lorentzian difference (LD) analysis quantifies the APT-weighted CEST effects through the Lorentzian fitting of the signal in a full *Z*-spectrum [16]. This method requires measurements at multiple off-resonance frequencies, inherently increasing acquisition times, but is presumed to provide a more accurate representation of the signal contributions originating from amides in mobile proteins and peptides [17–19]. This research aims to quantify the volumetric impact of integrating APT-weighted imaging into GTV delineation of glioblastoma, and to compare the use of APT-weighted MTR_asym_ and LD maps for target delineation.

## Methods

### Study participants

This study was approved by the Medical Ethics Review Committee of the Erasmus MC, Rotterdam, The Netherlands, and was performed in accordance with the Declaration of Helsinki. Adult patients diagnosed with glioblastoma and scheduled for radiotherapy with a total dose of 60 Gy or 40.05 Gy were eligible for inclusion. The diagnosis of glioblastoma was confirmed through pathology and molecular analysis following resection or biopsy, and made in accordance with the 2021 WHO classification of tumors of the central nervous system [20]. Patients who were referred for reirradiation or who had prior irradiation of the head-and-neck region were excluded. After providing written informed consent, recruited patients underwent an extended treatment planning MRI scan prior to radiotherapy. Radiation treatment adhered to clinical standards, relying exclusively on the structural MRI scans, and employing a 15-mm CTV-margin.

### MRI acquisition

Image acquisition prior to radiotherapy was performed on a 3-T hybrid positron emission tomography—PET/MRI scanner with a 40-channel head coil (SIGNA, GE Healthcare, Chicago, ILL, USA). As part of the clinical brain tumor imaging protocol, patients were imaged with the following sequences:

- unenhanced T1-weighted (three-dimensional fast spoiled gradient-echo, repetition time 6.3 ms, echo time 2.1 ms, voxel size 1 × 1 × 1 mm^3^);
- T2-weighted FLAIR (repetition time 7,600 ms, echo time 130 ms, voxel size 1.2 × 1.2 × 1.6 mm^3^);
- T2-weighted (repetition time 4,490 ms, echo time 145.7 ms, voxel size 0.6 × 0.6 × 3.0 mm^3^); and
- Contrast-enhanced T1-weighted (three-dimensional fast spoiled gradient-echo, repetition time 5.6 ms, echo time 2.3 ms, voxel size 0.9 × 0.9 × 1.6 mm^3^) after intravenous injection of Gadovist (solution 1 mmol/mL, dose 7.5 mL, and flow rate 5 mL/s).

For this research, the treatment planning MRI protocol was extended with a three-dimensional snapshot CEST sequence [21], acquired before administration of gadolinium-based contrast agent, with the following parameters: root mean square B_1_ power 1.5 µT; repetition time 6.6 ms; echo time 1.1 ms; the number of slices ≥ 16; voxel size 1.7 × 1.7 × 3 mm^3^; matrix 128 × 128; acceleration factor 3; flip angle 6°; frequency offsets ± 100, ± 50, ± 10, ± 8, ± 6, ± 5, ± 4, ± 3.5, ± 3, ± 2.5, ± 2, ± 1.5, ± 1.2, ± 1, ± 0.8, ± 0.5, and ± 0.25, 0 ppm. In addition, a reference image was acquired with saturation pulses at 300 ppm, capturing the equilibrium magnetization. For each patient, the field of view was manually adjusted by adapting the number of slices to cover the entire GTV at a minimum. The acquisition time of the CEST image series ranged between 4:38 min:s and 5:07 min:s; consequently, the duration of the standard clinical protocol plus the CEST sequence ranged between 20:45 min:s and 21:14 min:s.

### Image processing

For each patient, the CEST image series was motion corrected by linearly registering each image within the series to the 6 ppm image (mcflirt [22], FMRIB Software Library (FSL) v6.0.7, Oxford, UK). Thereafter, a brain mask was created from the 6 ppm image (HD-BET [23]), and applied to the motion-corrected CEST image series to perform brain extraction. Noise reduction was performed via non-linear filtering (SUSAN [24], FSL v6.0.7, Oxford, UK) and multilinear singular value decomposition (Tensorlab v3.0 toolbox [25]). To calculate the *Z*-spectra, the total image series was divided by the equilibrium magnetization image. Lorentzian fitting and voxel-wise B_0_ correction were done according to the post-processing methods described by Wu et al [19]. APT-weighted MTR_asym_ maps were calculated using the B_0_-corrected *Z* (3.5 ppm) and *Z* (-3.5 ppm) [15]. To correct spillover dilution, fluid suppression was integrated according to methods proposed by Schrüre et al [26]. The LD was computed by subtracting the *Z*-spectra from the fitted Lorentzian function; thereafter, the LD at 3.5 ppm was extracted to generate the APT-weighted LD map. To register the APT-weighted scans to the computed tomography scan, on which the conventional target delineations were delineated, the motion-corrected brain extracted 6 ppm CEST image was registered to the contrast-enhanced T1-weighted MRI scan (flirt [22, 27], FSL v6.0.7, Oxford, UK). The resulting transformation matrix was applied to the APT-weighted MTR_asym_ and LD maps to register these scans to the contrast-enhanced T1-weighted MRI scan. Thereafter, the transformation matrix derived from registering the contrast-enhanced T1-weighted MRI to the computed tomography scan for the clinical radiotherapy plan was utilized to register the APT-weighted MTR_asym_ and LD maps to the computed tomography scan.

### Target volume delineation

Delineation of the GTV and CTV adhered to the 2023 ESTRO-EANO guideline on target delineation and radiotherapy details for glioblastoma [3]. Generally, the GTV encompasses the resection cavity and residual contrast enhancement on contrast-enhanced T1-weighted images. The CTV expands the GTV with a 15-mm margin, which is adjusted for anatomical barriers like the falx cerebri or the tentorium cerebelli. To integrate APT-weighted imaging into target delineation, a biological tumor volume (BTV) was introduced that encompassed presumed tumor based on information derived from APT-weighted imaging. The BTV was defined by regions with hyperintense APT-weighted signal within residual contrast enhancement on contrast-enhanced T1-weighted MRI or peritumoral parenchymal hyperintensity on T2-weighted or T2-weighted FLAIR MRI. For each patient, the BTV was computed on the APT-weighted MTR_asym_ map (BTV_MTRasym_) and the APT-weighted LD map (BTV_LD_). Automatic segmentations of residual contrast enhancement and peritumoral parenchymal T2-weighted and T2-weighted FLAIR hyperintensities were created with HD-GLIO v2.0 [28, 29]. Regions with hyperintense APT-weighted MTR_asym_ or LD signal were identified in two steps: First, the white matter was automatically segmented on unenhanced T1-weighted MRI (fast [30], FSL v6.0.7, Oxford, UK) and registered to the contrast-enhanced T1-weighted MRI (flirt [22, 27], FSL v6.0.7, Oxford, UK) and computed tomography scan. By taking the white matter segmentation of the hemisphere without the primary tumor site, a segmentation of the contralateral normal-appearing white matter (cNAWM) was derived. Second, patient-specific thresholds were calculated for both the APT-weighted MTR_asym_ and LD maps *via* the Eq. 1 [18]:

1 $${S}_{{{\rm{APT}}},{thr}}\ge {\mu }_{{{\rm{APT}}},{cNAWM}}+2\times {\sigma }_{{{\rm{APT}}},{cNAWM}}$$

where *S*_APT,thr_ is the patient-specific threshold to identify hyperintense APT-weighted signal, µ_APT,cNAWM_ is the average APT-weighted signal intensity in the cNAWM, and σ_APT,cNAWM_ is the standard deviation of the APT-weighted signal intensities in the cNAWM. As imperfect motion correction for APT-weighted MTR_asym_ may yield erroneously high signal intensities in certain voxels, these particular voxels, defined as outliers, were intentionally excluded from the patient-specific threshold calculation to safeguard its validity. Outliers were identified *via* the Eq. 2:

2 $${S}_{{{\rm{MTRasym}}},{out}}\ge {Q3}_{{{\rm{MTRasym}}}}+1.5\times {{{\rm{IQR}}}}_{{{\rm{MTRasym}}}}$$

where *S*_MTRasym,out_ is the signal intensity cutoff for outliers, *Q3*_MTRasym_ is the upper quartile of the signal intensities in the brain segmentation on the APT-weighted MTR_asym_ map, and IQR_MTRasym_ is the interquartile range of the signal intensities in the brain segmentation on the APT-weighted MTR_asym_ map.

**Fig. 1:**
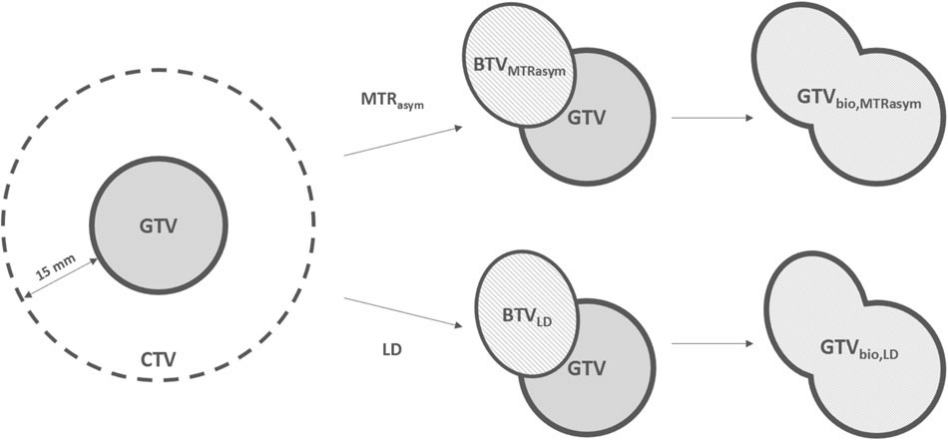
Target volume definition. Based on the APT-weighted MTR_asym_ and LD map, semi-automatically contoured, threshold-based BTV_MTRasym_ and BTV_LD_ were delineated. Thereafter, the fusion of the BTVs with the GTV defined the GTV_bio,MTRasym_ and GTV_bio,LD_. For abbreviations, see the “Abbreviations” section

Initial delineations of the BTV_MTRasym_ and BTV_LD_ were constructed by thresholding voxels with hyperintense APT-weighted MTR_asym_ and LD signal, respectively, within contrast-enhancing tumor and peritumoral parenchymal hyperintensity on T2-weighted and T2-weighted FLAIR images. Thereafter, components smaller than 1 mL were removed and smoothing was performed to generate the final BTVs. The final BTV_MTRasym_ and BTV_LD_ were added to the conventional GTV to define a biological GTV (GTV_bio_) based on APT-weighted MTR_asym_ (GTV_bio,MTRasym_) and LD (GTV_bio,LD_), respectively. Hence the GTV_bio_ comprises both anatomical information derived from structural MRI and biological information derived from APT-weighted imaging. Figure 1 shows a schematic overview of the target volumes generated in this study.

### Statistical analysis

The volumes of the conventional GTV, GTV_bio,MTRasym_, GTV_bio,LD_, and CTV were compared through a Wilcoxon signed-rank test. The difference between the use of APT-weighted MTR_asym_ and LD maps for target delineation was explored through a comparison of the BTV_MTRasym_ and BTV_LD_: A Wilcoxon signed-rank test was performed to evaluate the volume disparity between the BTVs. Additionally, the spatial similarity was explored by computing the Dice similarity coefficient.

## Results

### Study participants

Between June 2023 and December 2023, ten patients with glioblastoma were included in this pilot study. One patient was post-hoc excluded from analysis as the patient did not proceed with radiation treatment due to rapid clinical deterioration. The demographic and tumor characteristics of the patients included in the analysis are shown in Table 1.

**Table 1:** Patient and tumor characteristics

Patient	Sex	Age (years)	Tumor location	Extent of resection	MGMT promotor methylation status	Radiation treatment
#1	Male	52	Right	Partial	Methylated	30 × 2 Gy
#2	Male	51	Left	Partial	Unmethylated	30 × 2 Gy
#3	Male	64	Left	Partial	Unmethylated	30 × 2 Gy
#4	Male	63	Right	Partial	Methylated	30 × 2 Gy
#5	Male	66	Right	Gross total	Methylated	30 × 2 Gy
#6	Male	49	Left	Gross total	Unmethylated	30 × 2 Gy
#7	Male	57	Left	Gross total	Methylated	30 × 2 Gy
#8	Female	60	Right	Partial	Unmethylated	15 × 2.67 Gy
#9	Female	63	Bilateral	Partial	Unmethylated	15 × 2.67 Gy

*MGMT* O^6^-methylguanine-DNA-methyltransferase

### Volumetric analysis of the GTV, GTV_bio_, and CTV

**Fig. 2:**
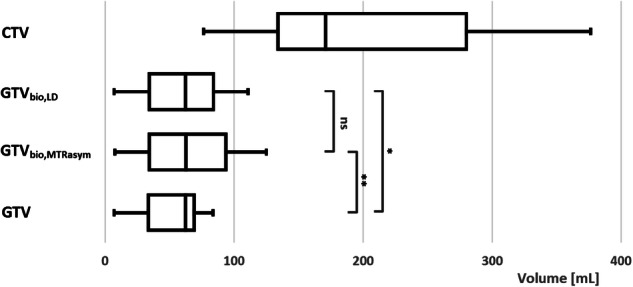
Box plot of the target delineation volumes. ns, not significant; *p < 0.05; **p < 0.01. For abbreviations, see the “Abbreviations” section

**Fig. 3:**
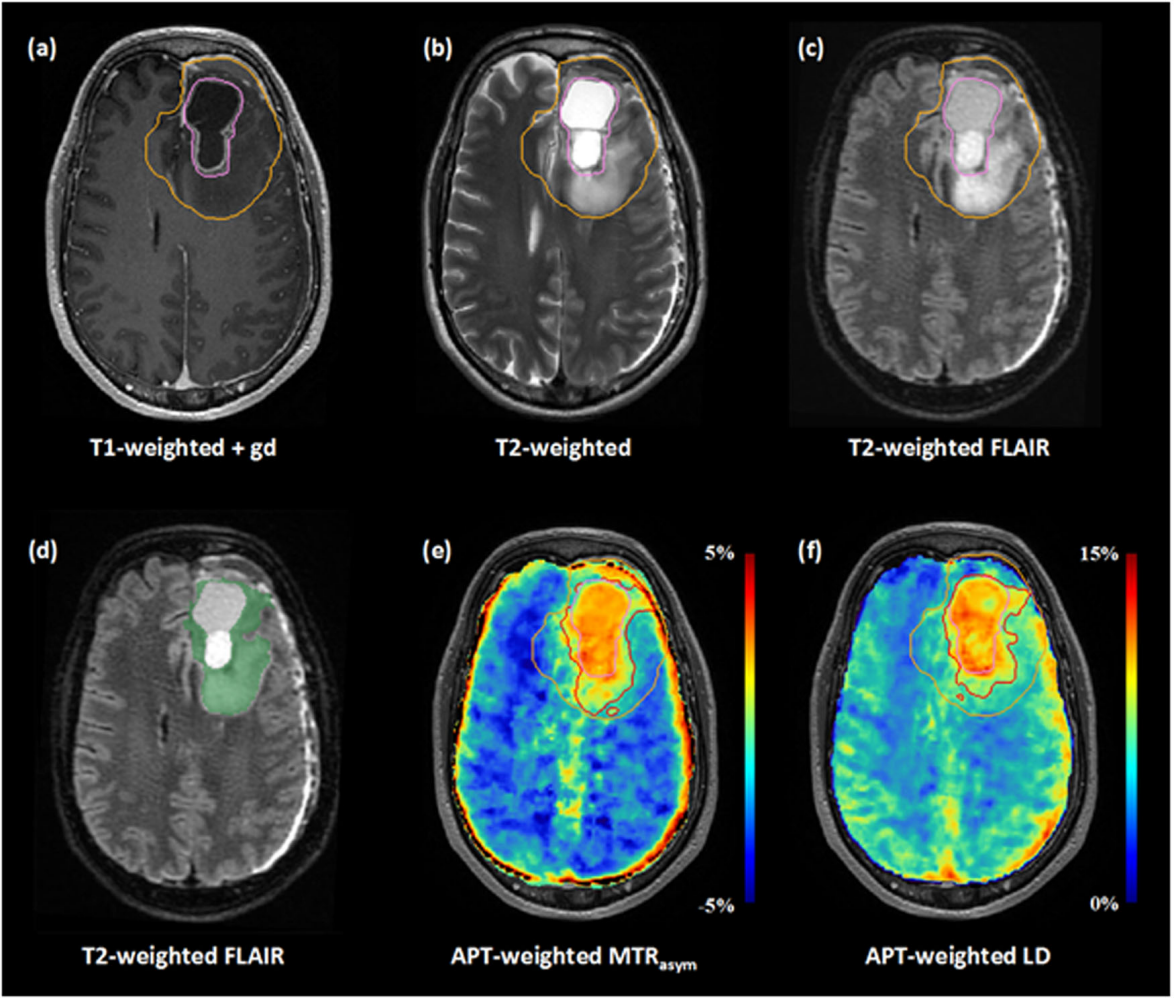
Target delineations of patient #2. The top row shows the conventional GTV (pink contour) and CTV (orange contour) on structural MRI (**a**–**c**). The automatic segmentation of residual contrast enhancement and peritumoral T2-weighted and T2-weighted FLAIR hyperintensities is shown as a green overlay on T2-weighted FLAIR MRI (**d**). Note that the resection cavity is not included in this segmentation. The conventional GTV (pink contour) and CTV (orange contour), as well as the GTV_bio,MTRasym_, and GTV_bio,LD_ (red contours), can be seen on the APT-weighted MTR_asym_ (**e**) and LD (**f**) map. Compared to the conventional GTV, the GTV_bio,MTRasym_, and GTV_bio,LD_ demonstrate an increase in volume of 81.2% and 52.4%, respectively. For abbreviations, see the “Abbreviations” section

The median patient-specific thresholds to define hyperintense APT-weighted signal for MTR_asym_ and LD were 0.48% (IQR: 0.15–0.58%) and 8.17% (IQR: 8.10–8.33%), respectively. Figure 2 shows a box plot of the conventional and APT-weighted target volumes. The comparative analysis revealed that both the GTV_bio,MTRasym_ and GTV_bio,LD_ were significantly larger than the conventional GTV, with a median increase in volume of 9.3% (*p* = 0.004) and 2.1% (*p* = 0.022), respectively. Additionally, the GTV_bio,MTRasym_ and GTV_bio,LD_ were significantly smaller than the CTV, with a median reduction in volume of 72.1% (*p* = 0.004) and 70.9% (*p* = 0.004), respectively. There was no significant difference in volume between the GTV_bio,MTRasym_ and the GTV_bio,LD_ (*p* = 0.164). The MRI scans and target delineations of an exemplary patient are presented in Fig. 3.

### Comparative analysis of the BTV_MTRasym_ and BTV_LD_

**Fig. 4:**
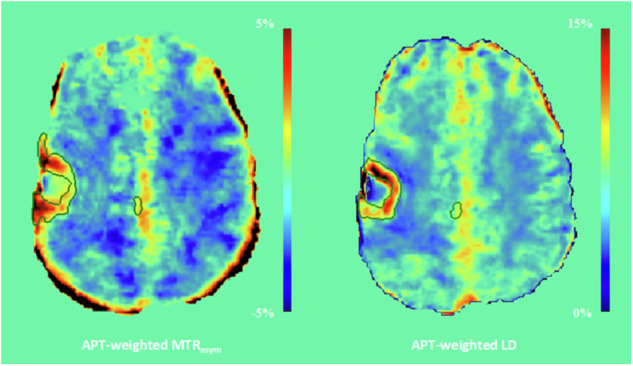
The APT-weighted MTR_asym_ and LD map and corresponding BTVs (green contour) of patient #4. The APT-weighted MTR_asym_ map shows voxels with relatively high signal intensities (> 5%) at the rim of the brain; these voxels did not exhibit proportionally high signal intensities in the APT-weighted LD map. For abbreviations, see the “Abbreviations” section

The median volumes of the BTV_MTRasym_ and BTV_LD_ were 26.2 mL (IQR: 6.0–38.9 mL) and 7.1 mL (IQR: 4.3–32.6 mL), respectively. Statistical analysis revealed no significant difference between the two BTVs (*p* = 0.074). The median Dice similarity coefficient between the two volumes was 0.59 (IQR: 0.17–0.71). In the APT-weighted MTR_asym_ maps, residual motion artifacts in the images acquired at 3.5 and -3.5 ppm resulted in relatively high signal intensities at the periphery of the brain; these elevated signal intensities were not visible on the APT-weighted LD maps. Consequently, in three of the nine patients, the BTV_MTRasym_ encompassed some of these voxels, while the BTV_LD_ did not. An example of this occurrence is shown in Fig. 4. The volumes of the GTVs, CTV, and BTVs of each individual patient are provided in Supplementary Table S1.

## Discussion

In this study, the potential of APT-weighted imaging for target delineation of glioblastoma is implied by the increase in volume of the GTV_bio,MTRasym_ and GTV_bio,LD_ in relation to the conventional GTV. The inability to reliably distinguish tumor infiltration from peritumoral edema on T2-weighted and T2-weighted FLAIR MRI is a major crux for target delineation of glioblastoma [3]. The introduction of APT-weighted imaging and the concept of a BTV may facilitate indirect visualization of tumor infiltration, enabling a reduction of the 15-mm CTV margin.

Notably, not all patients displayed a pronounced increase of the GTV after the integration of APT-weighted imaging. This observation may indicate the absence of tumor infiltration or tumor infiltration extending beyond the boundaries of contrast enhancement and T2-weighted or T2-weighted FLAIR hyperintensity. The latter scenario could occur, given that this study exclusively focused on elevated APT-weighted signals in these regions for defining the BTV. Alternatively, this observation may suggest that APT-weighted imaging by itself does not always adequately visualize tumor infiltration. It is important to note that this work does not yet determine if the BTV and GTV_bio_ truly encompass tumor infiltration. The rationale that T2-weighted and T2-weighted FLAIR hyperintense regions may partially reflect tumor infiltration, and that elevated APT-weighted signals may indicate tumor tissue in gliomas, is supported by previous studies [4, 11–13]. However, validation of the BTV and GTV_bio_ is an important next step that has yet to be undertaken. This step could be achieved through recurrence pattern analysis, which examines the coverage of future tumor recurrence by the GTV_bio_. The site of tumor recurrence, in hindsight, provides information on the location of tumor infiltration, thereby offering insights into the potential of a GTV_bio_ to include tumor infiltration. In addition, recurrence pattern analysis may shed light on the appropriate magnitude of the CTV margin around the GTV_bio_. We acknowledge that, to some extent, the comparison between the GTV_bio_ and conventional CTV in this work is premature. The significantly smaller GTV_bio_, however, adds strength to the hypothesis that the 15-mm CTV margin includes large amounts of presumably healthy tissue. While validation of the APT-weighted signal through recurrence pattern analysis is essential for evaluating the potential of APT-weighted imaging for improved target delineation, the results in our pilot study demonstrate the feasibility and volumetric impact of integrating APT-weighted imaging into target delineation of glioblastoma.

The volumetric similarity between the BTV_MTRasym_ and BTV_LD_ implies that utilizing LD analysis rather than the traditional MTR_asym_ metrics for APT-weighted imaging does not offer an evident benefit for target delineation of glioblastoma. Nevertheless, from a theoretical perspective, quantification through APT-weighted LD mapping may be advantageous for the semi-automatic, threshold-based approach presented in this study: APT-weighted LD analysis may more accurately reflect the authentic signal contributions originating from amides in mobile proteins and peptides, as this metric is not affected by the nuclear Overhauser enhancement effect [17–19]. The APT-weighted signal in MTR_asym_ metrics, on the other hand, is inherently contaminated by this effect [31]. Moreover, MTR_asym_ may be more sensitive to residual motion artifacts after motion correction: The required subtraction of the images acquired at 3.5 ppm and -3.5 ppm gave rise to erroneously high signal intensities at the periphery of the brain. In our results, the fitting procedure for LD seemed more robust against these residual motion artifacts. Although there was no significant volume difference in our data, the larger BTV_MTRasym_ compared to the BTV_LD_ and moderate dice similarity between the volumes may be partially explained by an unintentional overestimation of the BTV, which was caused by elevated APT-weighted signal intensities originating from residual motion artifacts rather than tumor physiology. This concern is particularly relevant for tumors located close to the cortex, as can be seen in the exemplary patient in Fig. 4. The APT-weighted MTR_asym_ map of this patient shows voxels with high signal intensities at the rim of the brain; however, these same voxels do not exhibit proportionally elevated signal intensities on the APT-weighted LD map, resulting in a disparity between the BTV_MTRasym_ and BTV_LD_.

There were some limitations in this study. First, the analyses in this study were performed on a small sample size. The primary aim of this pilot study, however, was to explore the impact on the GTV delineation in glioblastoma after the integration of APT-weighted MTR_asym_ and LD maps. Enhanced visualization of glioma infiltration through APT-weighted image acquisition at 7 T has already been demonstrated [32]. This study extends these findings by showcasing its feasibility at clinical field strength, and warrants further investigation of APT-weighted imaging for target delineation of glioblastoma through recurrence pattern analysis in a larger sample size. Second, the BTV was restricted to regions with contrast enhancement, which is typically included in the conventional GTV, and peritumoral nonenhancing parenchymal abnormalities, presumed to be a mixture of edema and tumor infiltration [3, 33]. In this study, we did not include hyperintense APT-weighted signals beyond contrast-enhancing tumor and T2-weighted or T2-weighted FLAIR hyperintense regions in the BTV. It is crucial to acknowledge that this approach might overlook tumor infiltration in normal-appearing brain tissue and necessitates future recurrence pattern analyses to assess elevated APT-weighted signals outside these regions. Finally, the field of view on APT-weighted imaging did not cover the entire brain in the craniocaudal direction, potentially resulting in an underestimation of the BTVs and GTVs_bio_. APT-weighted imaging of the entire brain is feasible at 3 T: acquisition of a full *Z*-spectrum, which enables both APT-weighted LD and MTR_asym_ mapping, requires approximately 6:30 min:s to 7:00 min:s on our system. Future work on recurrence pattern analysis should utilize APT-weighted imaging of the entire brain to minimize the risk of recurrences occurring outside the field of view of the APT-weighted map. Nevertheless, the significantly larger GTVs_bio_ in this study highlights the potential of APT-weighted imaging for target delineation of glioblastoma.

To summarize, in this study, the introduction of APT-weighted imaging yielded larger GTVs, suggesting visualization of tumor infiltration beyond the contrast-enhancing region and highlighting its potential for GTV delineation of glioblastoma. While there was no significant volumetric difference between the use of MTR_asym_ or LD to generate APT-weighted images, LD analysis might be preferred for target delineation due to its robustness against artifacts in the peripheral rim of the brain. Ultimately, the integration of APT-weighted imaging into radiotherapy planning may pave the way toward reducing the substantial 15-mm CTV margin, thus minimizing damage to healthy tissue while effectively irradiating the tumor. Our work provides the necessary foundation for the next step forward, which involves validating the GTV_bio_ through recurrence pattern analysis to assess its reliability and investigate if hyperintense APT-weighted signal beyond the boundaries of peritumoral nonenhancing parenchymal abnormalities should be included.

## Supplementary information

**Additional file 1:** **Supplementary Table 1.** Target volumes of each individual patient [mL].

## Data Availability

The datasets used and/or analyzed during the current study are available from the corresponding author upon reasonable request.

## Abbreviations

APT: Amide proton transfer
BTV: Biological tumor volume
BTV_LD_: Biological tumor volume based on the amide proton transfer-weighted Lorentzian difference map
BTV_MTRasym_: Biological tumor volume based on the amide proton transfer-weighted magnetization transfer ratio asymmetry map
CEST: Chemical exchange saturation transfer
cNAWM: Contralateral normal-appearing white matter
CTV: Clinical target volume
FLAIR: Fluid-attenuated inversion recovery
FSL: FMRIB software library
GTV: Gross tumor volume
GTV_bio_: Biological gross tumor volume
GTV_bio,LD_: Biological gross tumor volume based on the amide proton transfer-weighted Lorentzian difference map
GTV_bio,MTRasym_: Biological gross tumor volume based on the amide proton transfer-weighted magnetization transfer ratio asymmetry map
IQR: Interquartile range
IQR_MTRasym_: Interquartile range of the signal intensities in the brain segmentation on the amide proton transfer-weighted magnetization transfer ratio asymmetry map
LD: Lorentzian difference
MRI: Magnetic resonance imaging
MTR_asym_: Magnetization transfer ratio asymmetry
