# Supplementary material for: Amide proton transfer-weighted CEST MRI for radiotherapy target delineation of glioblastoma: a prospective pilot study
Source: Eur Radiol Exp. 2024 Oct 30;8:123. doi: 10.1186/s41747-024-00523-4 (PMC11525355; doi:10.1186/s41747-024-00523-4)
Supplement: Supplementary file 1 — Additional file 1: Supplementary Table 1. Target volumes of each individual patient [mL]. [file 41747_2024_523_MOESM1_ESM.pdf]

Amide proton transfer-weighted CEST MRI for radiotherapy target delineation of glioblastoma: a prospective pilot study

ELECTRONIC SUPPLEMENTARY MATERIAL

SUPPLEMENTARY TABLE 1

Target volumes of each individual patient [mL]

Patient	GTV	$GTV_{MTRasym}$	GTV_{LD}	CTV	$BTV_{MTRasym}$	BTV_{LD}
#1	33.65	34.18	34.35	145.02	5.33	7.11
#2	68.96	124.98	105.08	248.58	99.95	77.31
#3	46.31	52.28	50.51	134.00	26.45	12.46
#4	64.77	70.76	70.66	279.92	38.01	32.69
#5	6.93	7.54	6.93	76.42	2.60	0.00
#6	62.19	62.70	62.25	171.10	13.84	5.77
#7	30.48	32.82	30.48	104.88	6.04	0.00
#8	70.74	101.11	110.91	376.13	71.35	83.92
#9	83.59	93.47	83.91	334.84	26.21	4.30
